# Foundational Engineering of Artificial Blood Vessels’ Biomechanics: The Impact of Wavy Geometric Designs

**DOI:** 10.3390/biomimetics9090546

**Published:** 2024-09-10

**Authors:** Galip Yilmaz

**Affiliations:** Electronics and Automation Department, Bayburt University, Bayburt 69000, Turkey; galipyilmaz@bayburt.edu.tr; Tel.: +90-530-780-35-88

**Keywords:** artificial blood vessels, wavy geometric design, finite element method, hyperelastic materials, elasticity in artificial vessels, biomechanical engineering

## Abstract

The design of wavy structures and their mechanical implications on artificial blood vessels (ABVs) have been insufficiently studied in the existing literature. This research aims to explore the influence of various wavy geometric designs on the mechanical properties of ABVs and to establish a foundational framework for advancing and applying these designs. Computer-aided design (CAD) and finite element method (FEM) simulations, in conjunction with physical sample testing, were utilized. A geometric model incorporating concave and convex curves was developed and analyzed with a symbolic mathematical tool. Subsequently, a total of ten CAD models were subjected to increasing internal pressures using a FEM simulation to evaluate the expansion of internal areas. Additionally, physical experiments were conducted further to investigate the expansion of ABV samples under pressure. The results demonstrated that increased wave numbers significantly enhance the flexibility of ABVs. Samples with 22 waves exhibited a 45% larger area under 24 kPa pressure than those with simple circles. However, the increased number of waves also led to undesirable high-pressure gradients at elevated pressures. Furthermore, a strong correlation was observed between the experimental outcomes and the simulation results, with a notably low error margin, ranging from 19.88% to 3.84%. Incorporating wavy designs into ABVs can effectively increase both vessel flexibility and the internal area under pressure. Finally, it was found that expansion depending on the wave number can be efficiently modeled with a simple linear equation, which could be utilized in future designs.

## 1. Introduction

According to health statistics, cardiovascular diseases (CVDs) pose a significant concern for contemporary societies [1,2]. Extensive research has been conducted into new medical interventions, such as implants, devices, drugs, and other treatments [3,4,5]. This study specifically focuses on artificial blood vessels (ABVs) and highlights their importance in recent research, especially regarding mechanical compatibility and geometric design. ABVs are a preferred alternative when a patient’s native vessels are unsuitable for vascular surgery due to quality or availability problems. Although recent research on ABVs has significantly improved outcomes, further studies are needed to understand their long-term performance and mechanical compatibility better [6].

Ensuring that ABVs match the mechanical properties of young, healthy vessels is essential because mismatches can adversely impact blood circulation [7]. Studies have shown that natural arterial walls possess unique hyperelastic mechanical properties [8,9]. Increasing internal blood pressure can easily deform and enlarge the vessel diameter. At around 50% of strain [10], stiffer collagen fibers increase their resistance at a certain point, decelerating and then stopping the enlarging wall at a great resistance [11,12]. For the physiological success of the cardiovascular system, the artificially produced vessel must mimic the mechanical behavior of the native vessel. If it is too rigid, it will cause an abnormal pressure rise; if it is too soft, adequate blood transfer cannot be ensured. Achieving such fine-tuning is challenging due to the numerous material alternatives, production methods, and their countless combinations [13,14].

Recently, computer-aided design and analysis have been successfully employed to develop artificial vessels and address the challenges of precision production [15,16]. Multi-physics engineering simulation platforms have effectively studied various aspects of arterial grafts. These include using computational fluid dynamics to analyze patient-specific atherosclerosis, studying different material types for multi-layer arterial grafts, assessing the internal stress within the vessel, and examining various stages of aneurysm development [17,18,19]. Moreover, open-source software packages such as FEBio and Simvascular have gained significant traction in ABV research alongside commonly used general-purpose and commercial simulation platforms [20,21]. However, for these programs to be fully effective, additional time is required to overcome limitations in functionality and documentation. Ultimately, commercial or specific simulation programs offer significant advantages in fine-tuning the mechanical properties of designed artificial blood vessels (ABVs). They reduce the trial-and-error process and verify experimental results [20,21,22].

Another aspect to consider in ABV manufacturing is the material selection and the identification of proper manufacturing methods, which remains an intensely active area of research [6,23]. Among the assortment of materials that have gained widespread approval for clinical application are polytetrafluoroethylene (known as Teflon) and Dacron (a form of polyethylene terephthalate (PET) polymer fabric) [24,25]. Despite their clinical success, these materials intrinsically lack the essential flexibility required for ABVs when used in solid form. The primary approach has been using porous and loosely woven structures to address this limitation and enhance flexibility. However, these designs present challenges, are complex and costly to manufacture, and are prone to reliability issues [26,27,28]. On the other hand, materials characterized by hyperelasticity and extreme flexibility pose serious risks, such as aneurysm formation, rupture, and eventual failure over time [29,30,31].

Despite these shortcomings, employing a wavy cross-sectional architecture in vein construction has emerged as a promising strategy. This method has been supported by extensive research, demonstrating its ability to closely mimic natural blood vessels’ nonlinear tensile stress–strain relationship without compromising their mechanical strength or integrity [32,33,34,35]. However, a gap exists in the literature concerning the geometric intricacies of wavy ABVs. No comprehensive study has yet explored the geometric modeling of wavy-structured ABVs or examined their mechanical properties through both simulation and experimental methods. Specifically, the literature lacks information on the effects of increasing the number of waves and the appropriate modeling techniques.

The objective of this study is to design and analyze wavy geometries that enhance the functionality and flexibility of the artificial vessel. This study introduces a novel approach to enhancing the biomechanics of artificial blood vessels through wavy geometric designs, explored through a combination of CAD and FEM simulations alongside physical testing. It demonstrates that varying the wave numbers can substantially increase the flexibility and internal capacity of the vessels under pressure. A new mathematical model developed here quantitatively predicts how these changes affect vessel performance, providing a valuable framework for future advancements in vascular graft design. First, the wavy geometry of the artificial vessel was formed using a CAD program with a continuous curve composed of two inward and outward-curved segments. Then, a mathematical model was developed to determine how the number of curves and curvature radii influenced the vessel’s internal area. Using this model, the internal area was kept constant for all samples, including a flat circular geometry without any curves. After determining the cross-sectional geometry details of the samples, all with the same internal area, the final samples were modeled using a CAD program, resulting in ten sample types: a flat sample without any curves and nine samples with wavy geometries, with curve numbers ranging from six to twenty-two. Next, the hyperelastic material properties of the samples were analyzed using an engineering simulation program. Physical samples were then produced, and their expansion under pressure was examined using a new experimental method developed for this study. The simulation results were compared with the experimental outcomes, and an easy-to-use mathematical model was created. This model demonstrated the potential flexibility gained by varying the number of waves in the structure.

## 2. Materials and Method

**Figure 1:**
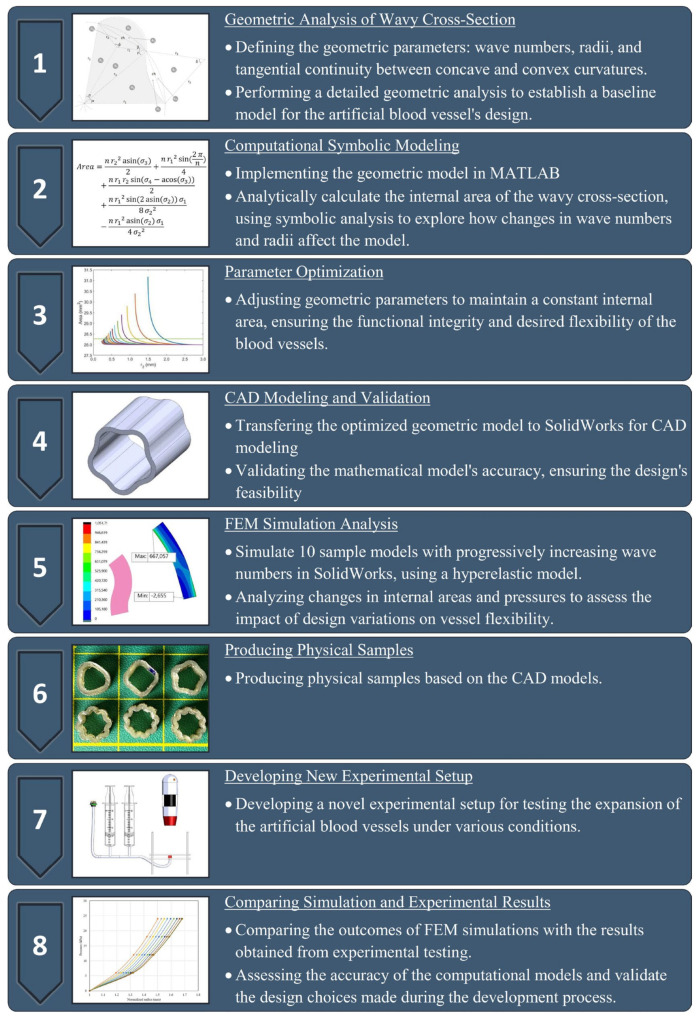
Schematic representation of the methodological approach adopted in the study to enhance the flexibility of artificial blood vessels. The flow chart shows the sequential steps from the initial geometric analysis of a wavy cross-section through computational modeling in MATLAB, parameter optimization, CAD modeling, and numerical validation in SolidWorks to the final simulation analysis and experimental testing.

Figure 1 shows the schematic representation of the methodological approach used in this study. The initial step involved a detailed geometric analysis of a wavy cross-section. Critical parameters such as wave numbers, radii, and tangential continuity between concave and convex curves were explicitly defined. A geometric model was developed using these parameters and implemented in a programming platform. Symbolic analysis was used to facilitate the analytical calculation of the internal area of the wavy section. This enabled the exploration of the impact of wave numbers and radii variations on the design. After completing the analytical phase, the geometric parameters were optimized to maintain a constant internal area, which is crucial for preserving the artificial blood vessels’ functionality and flexibility while keeping the same initial area despite the changing wave numbers. The refined model was then transferred to computer-aided design (CAD) software for modeling and numerical validation to confirm the accuracy of the mathematical model. Simulations were then conducted in a finite element analysis (FEA) module using a hyperelastic model to analyze changes in internal areas based on the increased inner pressure values. These simulations were crucial in assessing the impact of the proposed modifications on vessel flexibility. Furthermore, physical samples were fabricated based on the optimized CAD models and underwent pressure testing. A comprehensive comparison was then conducted between the simulation outcomes and experimental results to validate the proposed design approach and its potential to enhance the flexibility of ABVs.

### 2.1. Geometric Analysis of the Wavy Cross-Section and Sample Design

An initial reference sample was created to ensure that the final 3D models accurately reflect the intended design and maintain consistency across different specimen types. This sample represents vessels with a 6 mm inner diameter and a 0.5 mm wall thickness. It has a simple, flat, circular cross-section based on the literature [6,36] and serves as a baseline for comparing and modeling the other samples with curved geometries. The internal area of the other specimens with complex curved geometries can be accurately and consistently modeled by calculating it using the radius and the formula for the area of a circle based on the internal area of the reference geometry. This approach is fundamental to the study, ensuring all specimen types are modeled accurately.

Considering the alternative approach of maintaining equal perimeter and internal wall area, it becomes evident that while this task is mathematically simpler, it does not yield comparable samples in terms of blood-carrying capacity, especially when the number of waves significantly increases. In such cases, the internal area of the sample is substantially reduced, undermining the functional comparability of the models.

**Figure 2:**
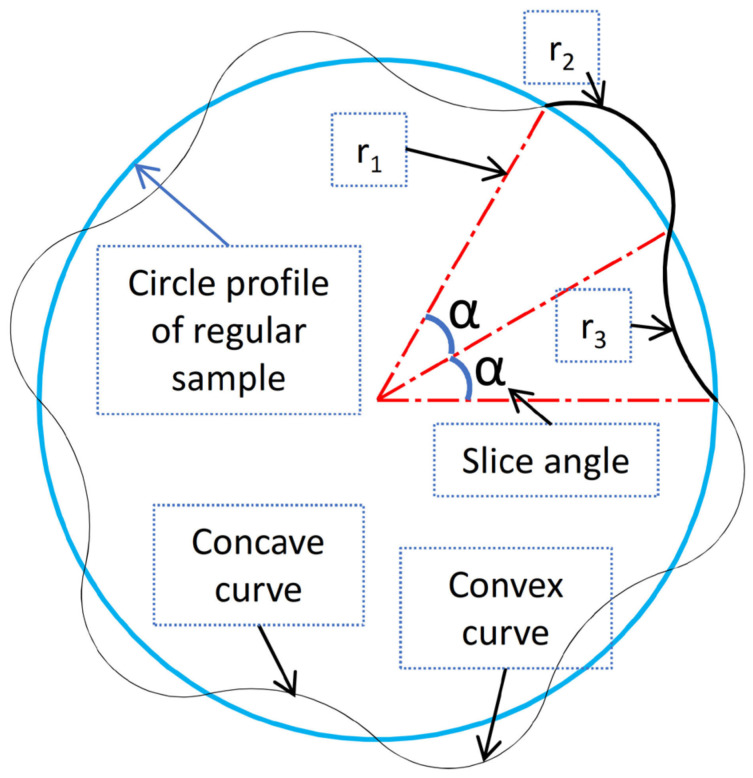
Inner profile geometry of the reference sample and a representative wavy pattern of the other samples.

After creating the reference sample, a systematic modeling approach was used to create nine additional samples based on the reference. The wall was modeled in these samples with a wavy structure to allow for more flexible material behavior. This adjustment aimed to address the material’s insufficient elasticity in vessels, which can increase blood pressure. The wavy vessel samples were designed to have the same internal area (lumen) and wall thickness (0.5 mm) as the reference sample. Determining the dimensions of the waves was a crucial control parameter to ensure the accuracy and functionality of the models. Figure 2 compares the reference sample’s circular geometry to the other models’ wavy geometry. The figure also includes variables relevant to the models’ construction, such as the radius of the base rings (r_1_) and the radii of concave and convex curves (r_3_ and r_2_, respectively), along with the angles (alpha) representing slice angles of the waves.

Table 1 lists the types of specimens created, including their names, the number of slices, and the dimensions of the base rings (r_1_). Following some trial and error, the radius of the wavy samples was slightly reduced by 0.5% to adjust for the changing area due to the wave radii alterations. This modification is essential to maintain consistency between the internal area and the reference geometry. The 0.5% reduction value was determined using the radii of both the flat ring regular sample and the first wavy sample. This specific reduction percentage was calculated when the derivative of the area changes of the wavy sample reached −1 based on r_2_. The choice of the derivative value at −1 is critical as it captures a response to the most significant change at an average point along the curve.

Due to the repetition of concave and convex pairs, all wavy samples exhibited an incrementally increasing number of segments by twos. Furthermore, designs did not include more than 22 segments or waves; beyond this number, the sample walls started to fold onto themselves.

### 2.2. Computational Symbolic Modeling

The objective was to develop an analytical model capable of calculating the internal area of the vascular model, accounting for varying radii and wave counts. Symbolic modeling was used in computer programming to analyze and design the complex wavy cross-sections of the ABVs. Symbolic modeling refers to a computational method that allows algebraic expressions to be manipulated and solved in symbolic form rather than numerically. This approach allows for precise analytical calculations, which is especially beneficial for complex geometric configurations. MATLAB^®^ (R2021a) was chosen because of its powerful capabilities and widespread use in scientific research. Using MATLAB’s Live Editor provided an interactive environment for scripting and output visualization, increasing the efficiency of symbolic computations. Symbolic Math Toolbox™ within MATLAB was used, which provides the ability to perform symbolic computations directly from the MATLAB command line by defining a particular data type as symbolic objects. The geometry was divided into smaller segments, and calculations for the area of each segment were performed symbolically using classical formulas for triangles and circular sectors.

### 2.3. Parameter Optimization

Upon reviewing Figure 2, it is evident that the wavy structure is defined by four parameters: r_1_, r_2_, r_3_, and α. However, only three parameters are necessary to define the structure fully. Among these, the radius of the concave curve, denoted by r_3_, has been chosen as the dependent variable. This means that a specific value for r_3_ is derived when the other three independent variables are inputted.

The convex curve (r_2_) radius has been selected as an optimization parameter to ensure the internal area is kept constant with the reference sample. Then, it determines the necessary r_2_ and the related r_3_. The intersection radius of the concave and convex curves (r_1_) has been kept constant for all samples. The angle of each slice is determined by dividing 360 degrees by the number of slices. The number of slices increases by two from 6 to 22 to calculate the necessary angles for each sample. A MATLAB code was used to calculate the parameters where each specimen’s internal area intersects with the internal area of the reference specimen for a specific r_2_ value.

### 2.4. CAD Modeling and Validation of Inner Area

**Figure 3:**
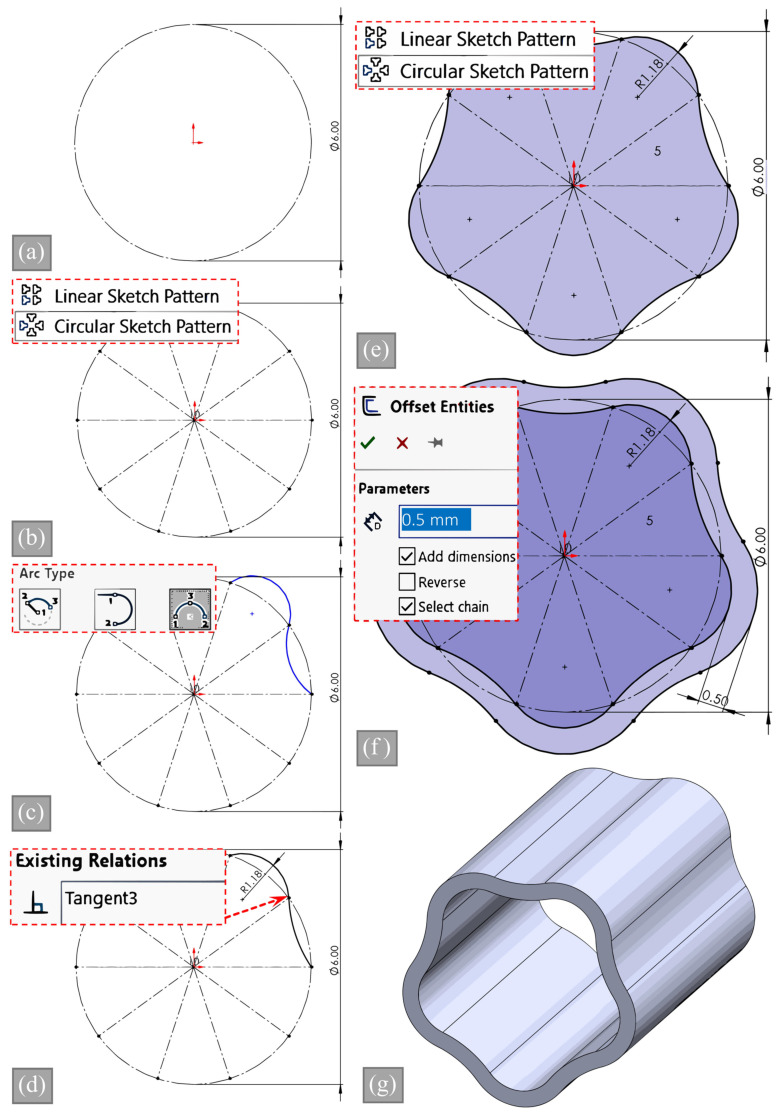
CAD modeling steps of the wavy profile: (**a**) reference circle, (**b**) circular sketch pattern for slices, (**c**) drawing inner and outer curves, (**d**) tangent relationships between two curves, (**e**) circular sketch pattern curve, (**f**) offset for 0.5 mm thickness, and (**g**) 3D extrusion of the section.

Creating systematic models of vessels with wavy structures is essential for other researchers to replicate modeling stages using different modeling programs. Figure 3 illustrates the modeling stages, which are described step by step. SolidWorks 2021, a widely used computer-aided design (CAD) software, was used to create the 3D models. The modeling process began with sketching a circle on the front plane, serving as the foundation for construction. A horizontal dashed line was then drawn from the circle’s center as a guide for creating symmetrical features. This line was duplicated using a circular sketch pattern with a number of slices corresponding to the desired features in the model, allowing for precise and uniform feature placement. To create the model’s waves, two separate curves were drawn, one facing outward and the other inward, which were then joined at one end and attached to an adjacent line on the other end. The curves were made tangent to ensure a seamless connection and to maintain the model’s symmetry. After completing the curves, a circle sketch pattern was used to create a closed loop, forming the basis of the model’s shape. The circular pattern was repeated for half of the total number of slices, ensuring the model was consistent and symmetrical. To add depth and dimension to the model, the offset entities feature of SolidWorks was utilized to create another loop positioned 0.5 mm outward from the original loop. Finally, the extruded boss feature was used to create a 3D solid model from the sketches and loops that were created. The result was a highly detailed and precise 3D model representing the intended design. After the CAD models of all samples were created, it was validated that the internal areas were the same using the “Measure” tool found under the “Evaluate” menu in the SolidWorks program.

### 2.5. FEM Simulation Analysis

The simulation module of SolidWorks 2021 software was used. The same material was used for each sample, which was 100E Universal Silicone, a one-part acetoxy silane-based RTV (room temperature vulcanizing) silicone from Akfix Chemistry, Istanbul, Turkey. The product, identified by the grade name “100E”, is a general-purpose silicone notable for its absence of additives and purity for experimental applications. The silicone is characterized by a Shore A hardness of 25, based on manufacturer data sheet (ASTM D2240), making it a typical hyperelastic material with high repeatability. This material was selected for the study primarily due to its wide availability, making it a readily accessible choice for research purposes. In addition, its inherent hyperelastic properties facilitate the study of materials under conditions that demand significant elasticity and durability.

The Mooney–Rivlin hyperelastic material model was readily available in SolidWorks. This model was preferred due to its widespread use and high accuracy. The Mooney–Rivlin model is a hyperelastic material model that describes the nonlinear elastic behavior of polymers, especially rubber-like materials. It utilizes strain energy functions that depend on material constants to describe materials under deformation. These constants characterize the material’s response to stress and strain, offering insights into its behavior under various conditions. The model’s versatility allows it to describe various mechanical properties, from simple to complex strain–stress relationships. Detailed model information can be found in the relevant literature [12,37,38]. Equation (1) below represents the Mooney–Rivlin model’s five-parameter version.

(1) $$w=A\left(J_{1}-3\right)+B\left(J_{2}-3\right)+\left(½\right)K{\left(J_{3}-1\right)}^{2}+C\left(J_{1}-3\right)\left(J_{2}-3\right)+D{\left(J_{1}-3\right)}^{2}+{E(J_{2}-3)}^{2}$$

Here, *w* represents the strain energy and the material constants *A*, *B*, *C*, *D*, and *E*, which are used in the equation and are determined through experimental data. The relevant material constants were obtained from the manufacturer and are listed in Table 2. These values were entered into the SolidWorks material library, and the custom model was created.

The simulation setup was designed as follows: (I) First, a representative, smaller section was extracted from the vascular cross-section geometry by slicing it with a plane through the midpoint of both the convex and concave sections. This resulted in a section that combines half of the convex portion and half of the concave portion. For the S0 sample, a section was taken at a 45-degree angle since there were no concave and convex sections for this sample. (II) Next, the simulation was set up by selecting 2D simplification and nonlinear study options for the repeating section, with the plane strain option chosen for simplification. (III) Pressure was applied to the inner surface as a boundary condition, and roller/cylinder boundary conditions were added to the cut surfaces. (IV) During the material selection phase, choices were made based on a custom material model. A hyperelastic Mooney–Rivlin model was used, and specific material parameters were entered into the model. (V) Meshing was performed using a “Very Fine” setting with an element size of 0.02 mm. (VI) Other critical settings include the singularity elimination factor set to 1, time increment auto-stepping selected, large displacement formulation activated, control technique force chosen, and iterative technique as Newton–Raphson employed. Finally, the Newmark method was selected as the integration method.

### 2.6. Producing Physical Samples

**Figure 4:**
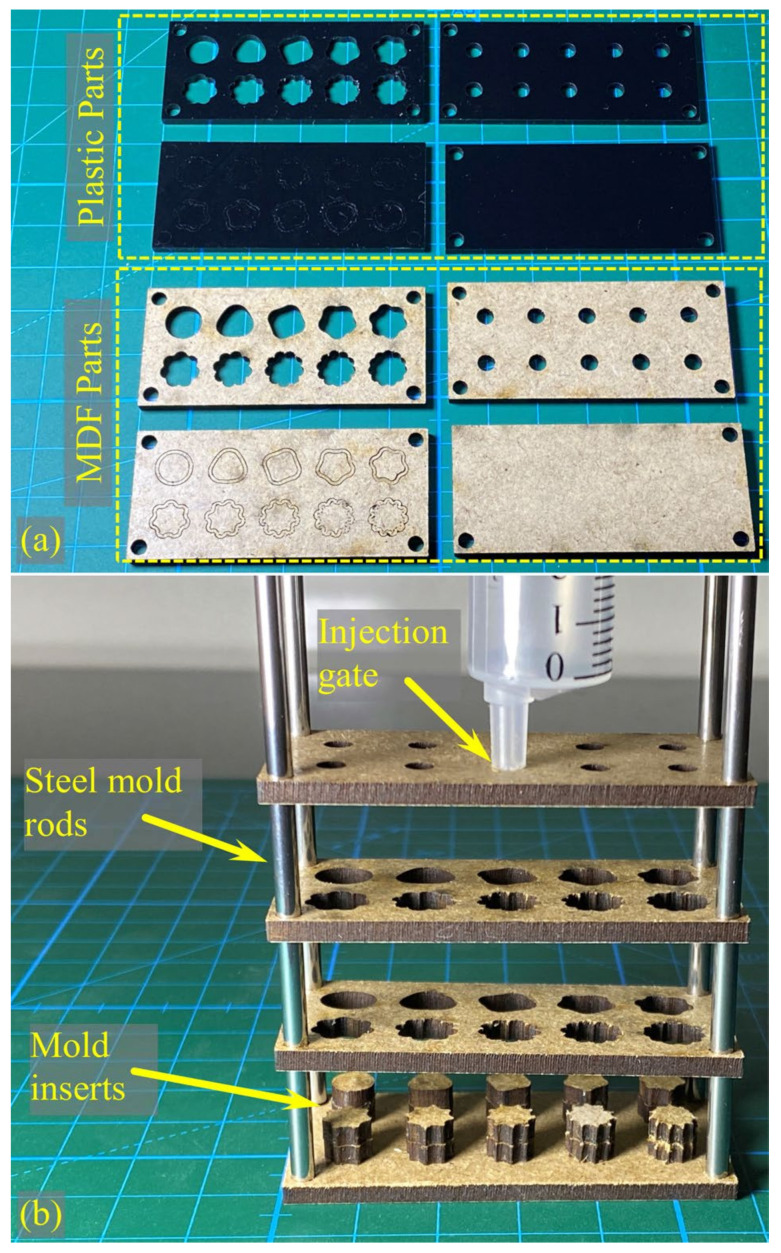
The mold parts used in the production of actual samples. (**a**) Two material types were tested for CNC laser-cut mold models: Poly(methyl methacrylate) (PMMA) plastic, commonly used in laser cutting, and MDF sheets. (**b**) An exploded view of the mold.

Figure 4 shows the mold parts used to produce the samples. Injection molding was chosen due to its effectiveness in overcoming air entrapment issues associated with the viscous nature of silicone. Two materials were tested for mold models cut using a CNC laser. The parts of these materials are shown in Figure 4a. Two materials were used in the laser cutting process: Poly(methyl methacrylate) (PMMA) plastic and an MDF product. Upon visual inspection, it was discovered that precise cutting could not be achieved with the plastic material due to melting issues caused by the laser’s heat. This was due to the small and detailed nature of the samples. In contrast, the MDF product, being cellulose-based, could be cut precisely. Additionally, unlike plastic, it allows for air passage, producing samples without curing problems. Figure 4b displays the exploded view of the mold.

### 2.7. Developing a New Experimental Setup

**Figure 5:**
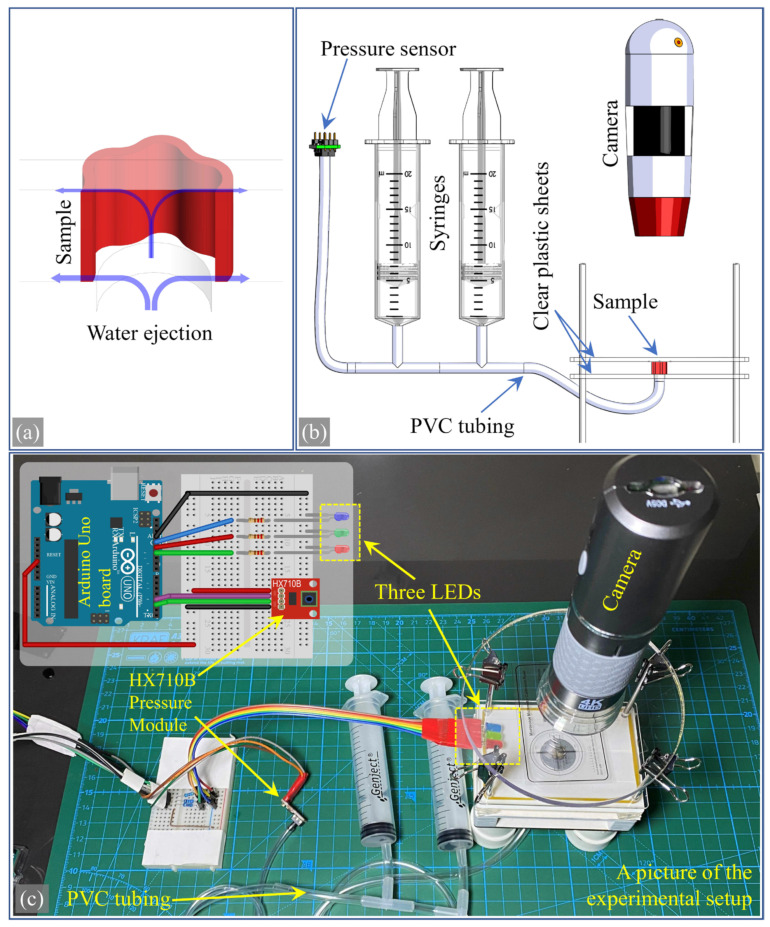
Experimental setup: (**a**) schematic picture showing the placement of the sample between two plates and the water outlets, (**b**) a schematic view detailing the application of water pressure and the observation method via a wireless camera, (**c**) a picture of the experimental setup and Arduino circuit with Three LEDs and pressure sensor.

A test setup designed for this study was developed to measure the expansion of a vascular cross-section. This apparatus facilitates the measurement of the increase in the cross-sectional area of samples cut to short lengths (4 mm) under the influence of pressure. Figure 5 shows an image and a diagram of this setup. The vascular section was placed between two transparent PMMA plates, with water pressure applied from below through a tube connected to a hole leading into the vascular section. As seen in Figure 5a, water exits through the top and bottom planes where the sample is in contact with the plates, thus balancing the pressure.

The schematic view in Figure 5b illustrates how the system operates. The expansion of the vascular section was observed from above with a wireless camera. The images obtained from the camera were processed with the open-source image software ImageJ 1.53t to measure the values of the area’s expansion due to pressure. Calibration of the measurements was performed with a microscope micrometer calibration slide. A pressure sensor and two 20 mL syringes were interconnected with PVC tubing, and the pressure of the water expelled from the syringe was displayed in real time. While the number of syringes can be increased, two were sufficient for water capacity. A triple-LED method was used for this setup to determine whether the water pressure was at the correct value. Blue, green, and red LEDs were programmed with an Arduino Uno control board to represent low, desired, and high pressure. These three LEDs were placed within the camera’s field of view, as shown in Figure 5c, allowing real-time monitoring of whether the water was at the desired pressure. The circuit shown in the insert image of Figure 5c was created with the open-source software Fritzing Beta 0.9.3. The software loaded onto the control board simultaneously operated the LEDs and a pressure module based on the HX710B chip.

### 2.8. Comparing Simulation and Experimental Results

The simulations and experiments were compared using relative error as a metric to quantify differences on a percentage basis. Pressure levels of 0, 12, and 24 kPa were used as standards to assess the comparative expansion of the inner vessel regions under these conditions. The boundaries of these areas were initially marked in a binary format during the experiments. The Wand tool in ImageJ was used to define the inner areas accurately. The ROI manager was used to calculate the area defined by the Wand tool.

## 3. Results

### 3.1. Mathematical Modeling of Vessel Sections

**Figure 6:**
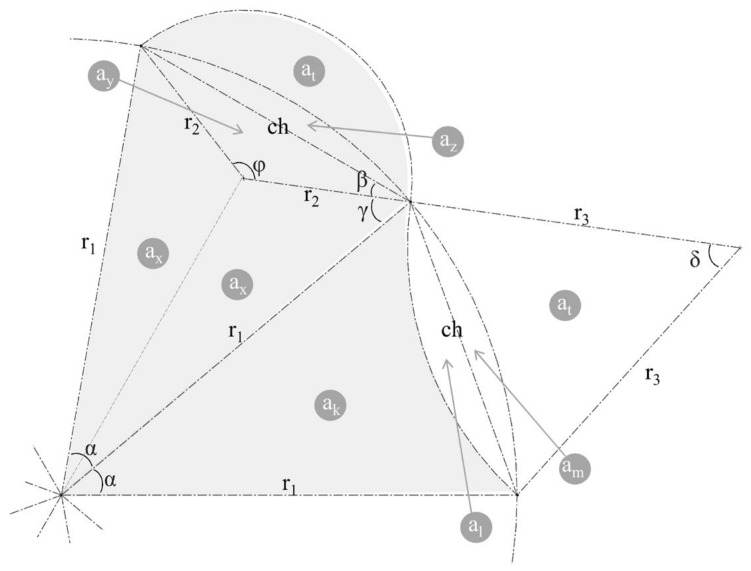
Geometry model of two slices; the letter “a” represents the area.

The modeling and engineering drawings of the vessel sections were performed using a systematic waveform drawing method, resulting in a geometry with both convex and concave sections. This geometry was modeled analytically to calculate the internal area. Figure 6 illustrates this model by selecting only two slices from the entire vessel section—since the others are repetitions of these slices. The purpose of this model is to allow analytical calculation of the internal area using the number of slices and radius parameters. The area calculation approach is based on individually calculating the area of the triangular sections within the slices and then summing these areas; the letter “a” with sub-indices represents these area sections.

The final analytical result obtained from the MATLAB symbolic analysis is shown in Equation (2). To make it more organized, σ symbols were used to create sub-equations for repeating parts of the equation, facilitated by the “live editor” in MATLAB. These equations are provided from Equations (3)–(7). MATLAB’s simplification function was also used to shorten the equation.

The area formula in Equation (2) was derived based on trigonometric functions. To test the numerical accuracy of this function, the area-finding function of a CAD program was used, and its accuracy was verified. The following section presents the measurements conducted for confirmation.

(2) $$Area=\frac{n\;{r_{2}}^{2}\;asin(\sigma_{3})}{2}+\frac{n\;{r_{1}}^{2}\;sin(\frac{2\;\pi }{n})}{4}+\frac{n\;r_{1}\;r_{2}\;sin(\sigma_{4}-acos(\sigma_{3}))}{2}+\frac{n\;{r_{1}}^{2}\;sin(2\;asin(\sigma_{2}))\;\sigma_{1}}{8\;{\sigma_{2}}^{2}}-\frac{n\;{r_{1}}^{2}\;asin(\sigma_{2})\;\sigma_{1}}{4\;{\sigma_{2}}^{2}}$$

(3) $$\sigma_{1}=cos(\frac{2\;\pi }{n})-1$$

(4) $$\sigma_{2}=cos(2\;\sigma_{4}-acos(\sigma_{3}))$$

(5) $$\sigma_{3}=\frac{\sqrt{2}\;r_{1}\;\sigma_{5}}{2\;r_{2}}$$

(6) $$\sigma_{4}=acos(\frac{\sqrt{2}\;\sigma_{5}}{2})$$

(7) $$\sigma_{5}=\sqrt{1-cos(\frac{2\;\pi }{n})}$$

### 3.2. Matching the Area of Samples

**Figure 7:**
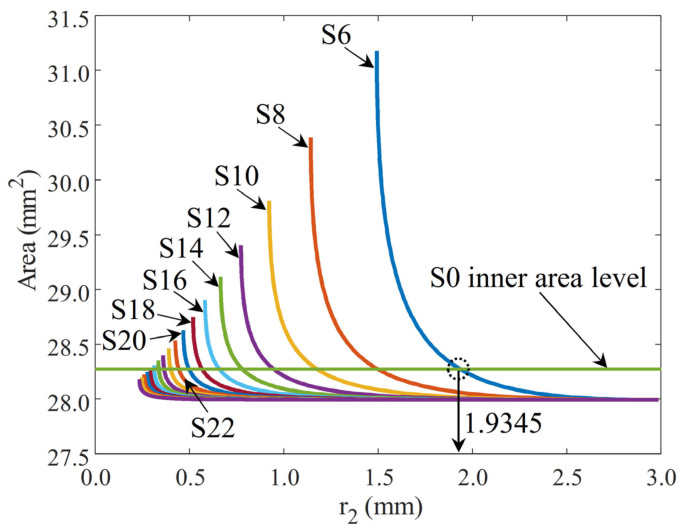
Area of sample geometries based on r_2_ values.

Equations and the code generated in MATLAB were used to produce the graph shown in Figure 7. The horizontal green line in the figure represents the reference sample (S0) area, which is 28.2743 mm^2^. This value was calculated analytically using the formula of the area of the circle with a 3 mm radius. For all other samples with a wavy structure, the area calculations were performed by entering the number of slices and base radius (r_1_) as independent variables into Equation (2), varying r_2_ values. The resulting area values are given in the graph. It should be noted that the r_3_ value is a dependent parameter. Due to geometric constraints, r_3_ adopts a certain value based on the chosen r_2_ value.

For each sample, the r_2_ values intersecting the horizontal area line of 28.2743 mm^2^ were found using numerical analysis in MATLAB. For example, in Figure 7, this intersection point for sample S6 is marked, and the r_2_ value is indicated. The r_2_ and r_3_ parameters determined for all samples are given in Table 3.

In Figure 7, each wavy sample exhibits a similar characteristic structure, showing an asymptotic rise at a certain r_2_ value. An increase in the r_2_ value reduces the internal area, converging towards a minimum area for all samples. In fact, an increase in the r_2_ value leads to the formation of a geometry resembling a flat circle that eliminates the wavy structure, which can be considered a predictable behavior. Conversely, decreasing the r_2_ value significantly increases the effect of the fold on the inner area. However, real geometry cannot be obtained after a certain peak point, so the area value is converted into a virtual numerical value. Thanks to the calculation of the effect of the wavy structure and r_2_ values of the samples on the area, it was ensured that the samples maintained the same geometric inner area, i.e., lumen.

**Figure 8:**
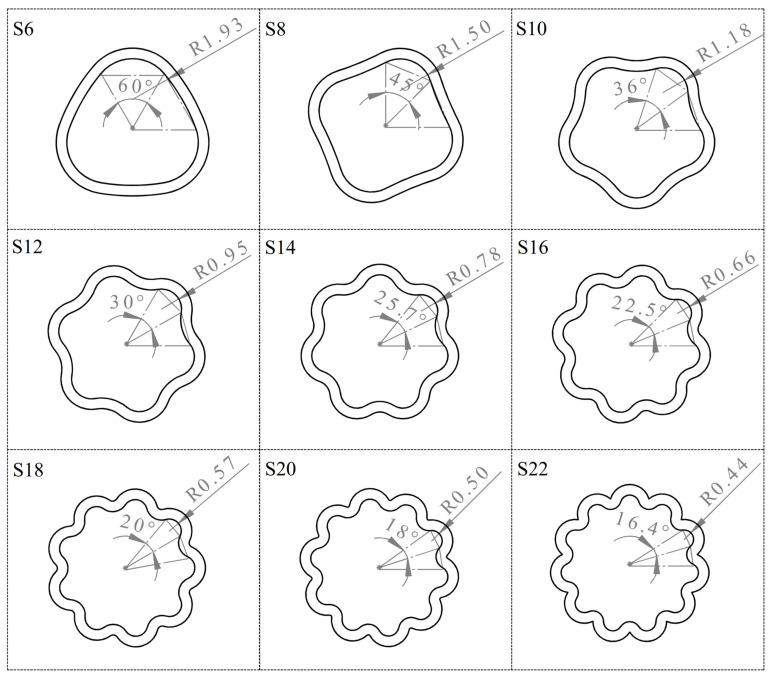
Technical drawing of wavy vessel cross-section profiles. The slice angles and r_2_ values can be seen for each sample in the technical drawings.

Final samples were created using the intersecting values for r_2_. The technical drawings of these samples are shown in Figure 8. Although the number of slices varies, the same inner area was maintained by selecting the correct r_2_ values. As seen from Table 3, increasing the number of slices decreased the r_2_ value. The area value, on the other hand, was accurately maintained thanks to the mathematical model developed in Equation (2). As an exceptional case, obtaining a concave shape in the S6 sample was impossible due to geometric constraints. This is because the diameter of the concave curve tends towards infinity, meaning it becomes a straight line. Therefore, both curves were chosen as convex for S6 as an exception.

Figure 8 shows the technical drawings of the samples identified parametrically. Notably, the initial values of the number of slices or bends create very different geometries. For example, a geometry with six slices has a smoothed triangular structure with rounded corners. Increasing the number of slices to eight leads to forming a square-shaped vein profile. It can be observed that with the number of slices set to ten, the wavy structure becomes more distinct. When the number of slices is increased to 22, the turning points of the outer curves overlap each other, resulting in a sharp structure. This folding would ultimately affect the mechanics of the vessel; hence, samples after S22 were excluded from the study.

### 3.3. Simulation Results and Hyperelastic Properties

**Figure 9:**
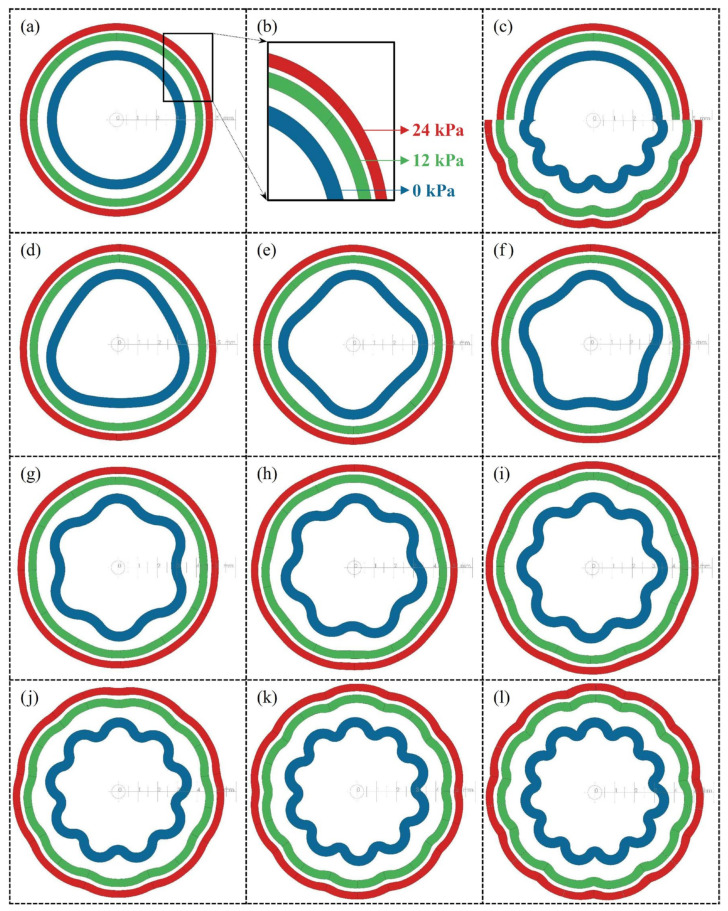
Blue rings represent the cross-sectional view with pressure at 0 kPa; green rings indicate expansion at 12 kPa, and red rings at 24 kPa. A ruler is placed from the center towards the left inside each set of nested rings, allowing the amount of expansion to be observed. (**a**) S0 sample, (**b**) colors of pressure levels, (**c**) the half-slices on the left side, combining S0 and S22 samples, are created to observe the difference in expansion, (**d**) S6, (**e**) S8, (**f**) S10, (**g**) S12, (**h**) S14, (**i**) S16, (**j**) S18, (**k**) S20, and (**l**) S22.

Figure 9 shows the SolidWorks simulation results. The expansions of the specimens were compared using three pressure levels (0, 12, 24 kPa) and are arranged in nested rings. At the high-pressure value (24 kPa), it can be observed that as the number of slices increases, the wavy structure on the outer surfaces of the specimens becomes more pronounced. In other words, as the number of sections decreases, it can be seen that the outer surface takes on a completely circular shape. The persistence of the wavy pattern despite expansion indicates significant stress differences within the material. In general, the impact of this behavior on the long-term performance of the material in practical applications, as well as its physiological effects, should be investigated in future studies.

**Figure 10:**
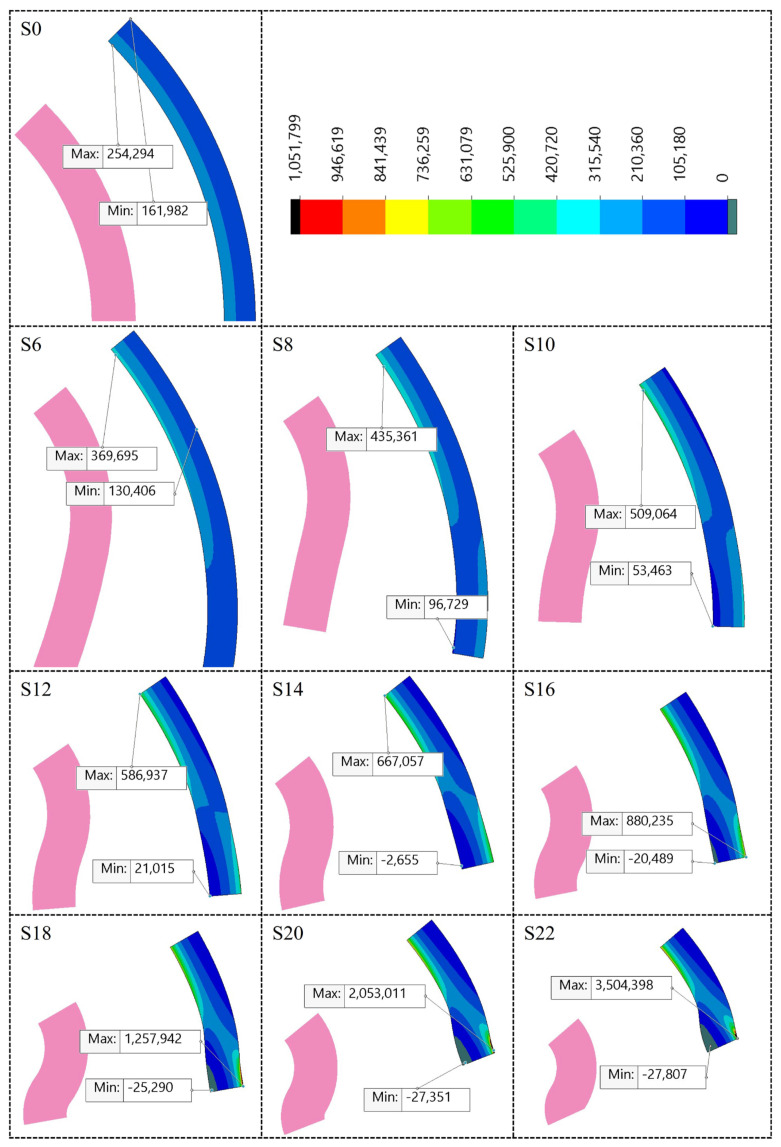
The simulation results and a color scale indicate the principal stress values at the 24 kPa inner pressure. The section shown in pink color represents the original cross-section prior to the application of pressure (0 kPa case).

To better observe the internal stress distribution, the stress contour plots of the samples with a segment created by the combination of half sections of concave and convex wavy structures are shown in Figure 10. The color map is fixed at a maximum point average of 1051 kPa, with higher values shown in black color. A value of 0 kPa was chosen for the minimum point, and areas of negative (compressive stress) are depicted in gray. The section shown in pink color represents the original cross-section prior to the application of pressure (0 kPa case). It can be seen that as the number of slices increases, the difference in stress distribution within the specimens increases. Minimum stress values are seen in the center of the concave curve starting with specimen S8. The maximum stress values are observed in the outward concavity starting with specimen S16. This indicates that a significant stress difference develops from the inner to the outer wall as the number of curves increases. It can be assumed that in a thin-walled structure, this situation would harm the long-term performance and reliability of the vessel. However, the trade-off with the flexibility it provides should be investigated in future studies.

### 3.4. Experimental Results and Their Comparison with Simulation Results

**Figure 11:**
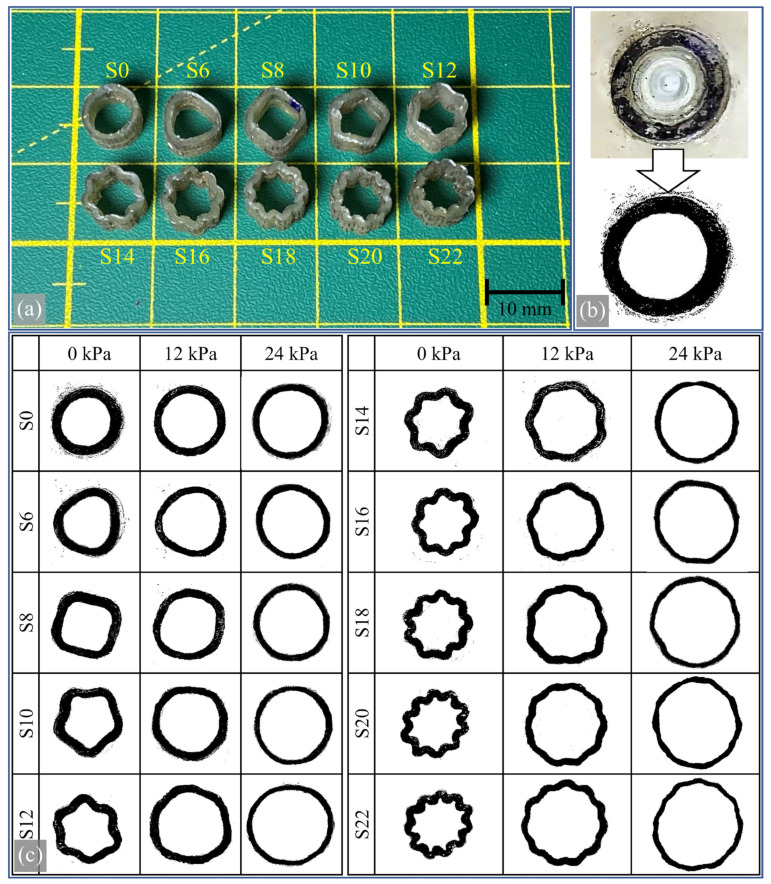
Processing the inner areas of the produced samples with an image processing program: (**a**) an image of the produced samples, (**b**) an image showing the transformation of the inner area with image processing, (**c**) changes in the inner areas of the samples with 0, 12, and 24 kPa pressure.

Figure 11 shows visuals of the compression tests performed on the produced samples. Figure 11a shows an image of the samples removed from the injection mold after curing. The visual inspection confirmed that the production was carried out without defects, such as air traps, and that the dimensions were as required. Figure 11b shows a representative image processed using ImageJ software. The sections of the samples facing the camera were permanently marked with a black marker to enhance the clarity of the image analysis. In addition, color discrepancies and visible areas within the section were manually painted black using ImageJ’s airbrush tool, while watermark-like shapes were painted white using the same method. This resulted in the images shown in Figure 11c. Upon visual inspection, it can be observed how the inner areas of the samples expand with increasing pressure. The waves of the specimens spread out under pressure, and all samples take on a similar shape, forming a circular structure. However, some bulges and irregular shapes were observed, disturbing the perfect ovality. For example, it can be observed that sample S22 at 24 kPa pressure becomes much more irregular in shape than sample S0 at the same pressure. The main reason for this irregularity can be attributed to the specimen’s lack of perfect geometric symmetry during production. Another reason may be the varying friction between the expanding section and the transparent plates at different locations and the inability to achieve a perfectly ideal surface during manufacturing. For example, areas with less friction deform more outward from the center, while areas with more friction remain closer to the center.

**Figure 12:**
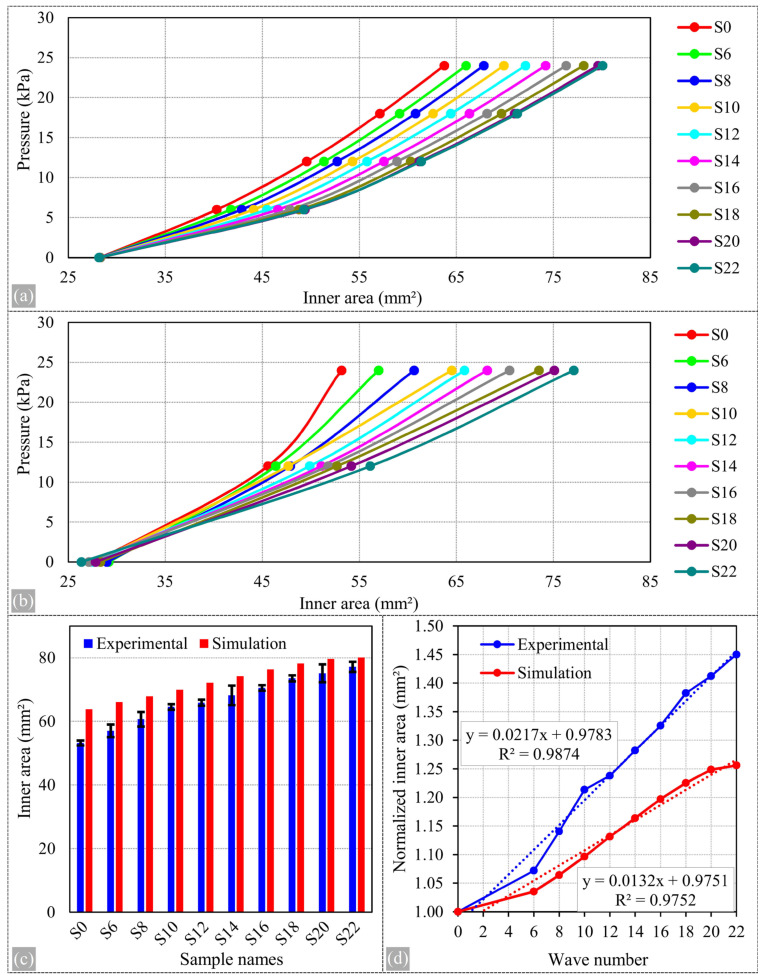
Graphs demonstrating the expansion of internal areas under pressure. (**a**) Graph showing pressure and internal area from simulation results. (**b**) Graph showing pressure and internal area from experimental results. (**c**) Comparison of experimental and simulation results for an internal pressure of 24 kPa. (**d**) Graph depicting normalized internal areas by the increasing number of waves for the pressure of 24 kPa from both experimental and simulation results.

To provide a quantitative analysis of the results, measurements of the internal areas of three different samples were taken, and the results were then compared to the simulation results in the graphs. Figure 12 shows the graphs of this comparison. Additionally, Table 4 compares the results for the areas obtained at 24 kPa and includes the corresponding error percentages. Figure 12a,b show the results of area calculations obtained from simulations and experiments, respectively, displayed on the same axis scale, one above the other. Overall, there is significant agreement between the experimental and simulation results. The maximum error percentage, calculated to be 19.88% for sample S0, decreases as the number of waves in the sample increases. The error percentage reduces to 3.84% for sample S22.

Table 4 and the bar charts in Figure 12c compare the expansion of the samples’ internal areas at 24 kPa pressure. The standard deviation values for the experimental results are also included in this graph. It is observed that the experimental results yield lower values compared to the simulation results. This discrepancy can be attributed to imperfections in the material model, the complexity of the hyperelastic nonlinear behavior, and the propensity of experimental dynamics to generate errors.

Another primary reason for this discrepancy could be attributed to the water pressure not expanding the sample to its maximum possible structure but instead allowing the sample to relax slightly during water escape. This issue is minimized in the S22 sample due to its longer cross-sectional line, which provides a greater capability to balance the pressure. Nevertheless, these conditions must be validated in future studies. It is also necessary to ascertain the compatibility of the hyperelastic model with real-world experiments and to verify whether the model accurately represents actual conditions.

As anticipated, simulation results tend to exhibit an ideal pattern, while experimental results display some irregular characteristics due to the imperfect geometries of the samples and experimental conditions. Figure 12d illustrates the increase in the internal areas of the samples at 24 kPa pressure according to the number of waves. The experimental results were normalized to their own S0 value (53.18 mm^2^), and the simulation results to their own S0 value (63.76 mm^2^). A linear fit was then applied to the resulting graph. The R-squared value for both experimental sets was found to be nearly identical, indicating that the increase in the number of waves can predict the extent to which blood pressure facilitates the expansion of the vessel’s internal area. A simple linear equation can describe this relationship. This study presents detailed analyses of the effects of pressure changes on the internal structures of the samples. Figure 12d, in comparison with the control sample, clearly shows how the increase in pressure interacts with the number of waves. These results allow us to assess the performance of the hyperelastic model under real experimental conditions, demonstrating the accuracy of the model and its potential for engineering applications.

## 4. Conclusions

This study focused on the design of wavy structures in manufacturing vascular grafts, a topic often addressed in practice but not systematically studied in the literature. Computer-aided engineering design methods and simulations were utilized, and the results were validated with physical samples to provide a foundation for researchers to use in their designs and similar studies. The findings of this study are outlined below:

- The creation of a wavy blood vessel cross-section required the investigation of numerous geometric parameters. A geometric approach incorporating both concave and convex curves was successfully created and modeled using symbolic mathematical tools in a computational environment.
- This analytical model was validated using CAD software to ensure that the internal area of the vessel cross-section (representing the lumen) remained constant across all samples as the number of waves increased. Additionally, technical drawings of the specimens, which are critical for repeatability, were generated for different wave numbers.
- A total of ten CAD models were subjected to internal pressure using a hyperelastic material model within a finite element method (FEM) module to investigate the expansion of the internal areas under varying pressure conditions.
- A unique experimental setup was developed to observe the expansion of the internal areas and the expansion of the waves under pressure. The results showed that the vessels’ flexibility effectively increased with the number of waves. For example, a sample with 22 folds had 45% more area under 24 kPa pressure than a sample with no waves. However, it was also found that increased wave numbers resulted in undesirably high-pressure gradients at higher pressures.
- A notable correlation was observed between experimental and FEM simulation results. The discrepancy was minimal, with a maximum difference of 19.88% decreasing to a mere 3.84% among sample types. This degree of precision is a significant achievement for a model exhibiting hyperelastic nonlinear behavior.
- The expansion of the internal area was found to be modeled by a simple linear equation, which provides a practical calculation method that researchers can use during the design phase. This equation demonstrates the contribution of increasing wave numbers to the expansion of the internal area.

Future studies should consider exploring three-dimensional wave patterns in the design of artificial blood vessels to potentially enhance both longitudinal and radial expansion. This advancement could provide a more comprehensive understanding of biomechanical properties under simulated physiological conditions. Investigating the use of various hyperelastic materials may also reveal insights into the adaptability and durability of these vessels under cyclic loading. It is proposed that advanced computational modeling and high-resolution finite element analysis be employed to optimize design for improved performance and safety, aiming to develop more effective and reliable vascular grafts suitable for clinical applications.

## Figures and Tables

**Table 1:** Designated sample names, slice numbers, and base radii.

Sample Name	Slice Numbers	r_1_ (mm)
S0	0	3.0000
S6	6	2.9850
S8	8	2.9850
S10	10	2.9850
S12	12	2.9850
S14	14	2.9850
S16	16	2.9850
S18	18	2.9850
S20	20	2.9850
S22	22	2.9850

**Table 2:** Material properties, including Poisson’s ratio and five material constants (ranging from the first to the fifth).

Property	Value	Unit
Poisson’s Ratio	0.49	N/A
First Material Constant (*A*)	36,958	N/m^2^
Second Material Constant (*B*)	−8783	N/m^2^
Third Material Constant (*C*)	−5907	N/m^2^
Fourth Material Constant (*D*)	42,613	N/m^2^
Fifth Material Constant (*E*)	2737	N/m^2^
Mass Density	1130	kg/m^3^

**Table 3:** Sample parameters based on slice number and r_2_ and r_3_ values. (* The value is from the analytical calculation.) (** As an exceptional case, obtaining a concave shape in the S6 sample was impossible due to geometric constraints, so both curves were chosen to be convex).

Sample Name	Slice Numbers	Slice Angle (deg)	r_1_ (mm)	r_2_ (mm)	r_3_ (mm)	Area (mm^2^)
S0	0	NA	3.0000	NaN	NaN	28.274 *
S6	6	60.00	2.985	1.934	9.034 **	28.274
S8	8	45.00	2.985	1.497	13.842	28.274
S10	10	36.00	2.985	1.176	3.413	28.274
S12	12	30.00	2.985	0.945	1.841	28.274
S14	14	25.71	2.985	0.779	1.228	28.274
S16	16	22.50	2.985	0.658	0.909	28.274
S18	18	20.00	2.985	0.566	0.718	28.274
S20	20	18.00	2.985	0.497	0.592	28.274
S22	22	16.36	2.985	0.443	0.505	28.274

**Table 4:** Inner areas from simulation and experimental results at the pressure of 24 kPa.

	S0	S6	S8	S10	S12	S14	S16	S18	S20	S22
Experimental areas (mm^2^)	53.18	57.00	60.65	64.53	65.85	68.18	70.49	73.53	75.10	77.12
Standard deviation	0.78	1.97	2.31	0.86	0.95	3.07	0.90	0.95	2.78	1.61
Simulation areas (mm^2^)	63.76	66.00	67.84	69.90	72.12	74.20	76.32	78.12	79.60	80.08
Error %	19.88	15.79	11.85	8.33	9.53	8.84	8.27	6.25	5.99	3.84

## Data Availability

All relevant data presented in the study are included in the article, further inquiries can be directed to the corresponding author.
